# Serum neurofilament light chain is a discriminative biomarker between frontotemporal lobar degeneration and primary psychiatric disorders

**DOI:** 10.1007/s00415-019-09567-8

**Published:** 2019-10-08

**Authors:** Kasper Katisko, Antti Cajanus, Olli Jääskeläinen, Aleksi Kontkanen, Päivi Hartikainen, Ville E. Korhonen, Seppo Helisalmi, Annakaisa Haapasalo, Heli Koivumaa-Honkanen, Sanna-Kaisa Herukka, Anne M. Remes, Eino Solje

**Affiliations:** 1 Institute of Clinical Medicine–Neurology, University of Eastern Finland, Yliopistonranta 1C, 1627, 70211 Kuopio, Finland; 2 Neuro Center, Neurology, Kuopio University Hospital, Kuopio, Finland; 3 Neuro Center, Neurosurgery, Kuopio University Hospital, Kuopio, Finland; 4 A.I. Virtanen Institute for Molecular Sciences, University of Eastern Finland, Kuopio, Finland; 5 Institute of Clinical Medicine–Psychiatry, University of Eastern Finland, Kuopio, Finland; 6 Department of Psychiatry, Kuopio University Hospital, Kuopio, Finland; 7 MRC Oulu, Oulu University Hospital, Oulu, Finland; 8 Research Unit of Clinical Neuroscience, Neurology, University of Oulu, Oulu, Finland

**Keywords:** Frontotemporal dementia, Frontotemporal lobar degeneration, Psychiatric disorders, Biomarker, Neurofilament light (NfL), sNfL

## Abstract

Due to the significant clinical overlap between frontotemporal lobar degeneration (FTLD) spectrum disorders and late-onset primary psychiatric disorders (PPD), diagnostic biomarkers reflecting the different underlying pathophysiologies are urgently needed. Thus far, elevated cerebrospinal fluid (CSF) levels of neurofilament light chain (NfL) have been reported in various neurological conditions. Furthermore, recent advancements in ultrasensitive analytical methods (e.g., single molecule array, Simoa) have enabled sensitive and less invasive NfL detection also from blood samples. In this study, we evaluated the potential of serum NfL (sNfL) as a diagnostic tool between FTLD and PPD. We analyzed sNfL levels with Simoa from 125 participants including patients from FTLD (*n* = 91) and PPD (*n* = 34) spectra. Our results show that sNfL levels are higher in the FTLD group compared to the PPD group as well as in separate clinical subtypes of FTLD compared to different psychiatric manifestations (i.e., mood or psychotic disorders). At single-subject level, discrimination between FTLD and PPD was possible with 80% sensitivity and 85% specificity (AUC = 0.850, 95% CI 0.776–0.923), and between behavioral variant frontotemporal dementia (bvFTD) and PPD with 79% sensitivity and 85% specificity (AUC = 0.830, 95% CI 0.732–0.908). These findings highlight the potential of sNfL as a discriminating biomarker for FTLD over PPD in patients with wide-ranging behavioral, psychiatric and cognitive symptoms.

**Electronic supplementary material:**

The online version of this article (10.1007/s00415-019-09567-8) contains supplementary material, which is available to authorized users.

## Introduction

The diagnosis of frontotemporal lobar degeneration (FTLD) and especially the behavioral variant frontotemporal dementia (bvFTD) subtype is often challenging, as the heterogeneous clinical manifestation may overlap not only with other neurodegenerative diseases but also with primary psychiatric disorders (PPD) [1–3]. Thus, biomarkers with a potential to accurately discriminate diseases with wide-ranging neuropsychiatric phenotypes are needed for early and correct diagnosis.

Neurofilament light (NfL), medium (NfM) and heavy (NfH) proteins have recently emerged as potential biomarkers for axonal damage in various neurodegenerative diseases, such as FTLD, Alzheimer`s disease (AD) and Parkinson`s disease (PD) [4]. The diagnostic potential of especially NfL is additionally supported by the fact that current ultrasensitive analytic platforms have enabled NfL detection from peripheral blood samples, providing a convenient and less invasive option for measuring diagnostic markers compared to CSF [5]. A recent study comprising 20 bvFTD patients and 50 psychiatric patients suggested that serum NfL (sNfL) may be used as a biomarker between bvFTD and psychiatric disorders [6].

The aim of this study was to compare sNfL levels in a clinical setting using a larger cohort comprising patients with adult-onset neuropsychiatric or cognitive symptoms, eventually diagnosed either as FTLD spectrum disorder or PPD.

## Materials and methods

### Study groups

All patients (FTLD and PPD) were examined at Kuopio University Hospital between the years 1998–2016 by a neurologist specialized in memory diseases. The PPD patients were diagnosed by a psychiatrist and neurodegenerative disorder was excluded from these patients by a neurologist. Patients (FTLD and PPD) underwent neurological and neuropsychological examination and brain imaging (magnetic resonance imaging (MRI), followed with positron-emission tomography (PET) or single-photon emission computed tomography (SPECT) if necessary), and they were classified according to the current FTLD diagnostic criteria [bvFTD, non-fluent variant primary progressive aphasia (nfvPPA) and semantic variant primary progressive aphasia (svPPA)] [7, 8]. FTLD patients that were diagnosed before 2011 were originally diagnosed according to the Neary 1998 criteria [9] and retrospectively evaluated to meet the Rascovsky or Gorno-Tempini criteria. For bvFTD, only patients fulfilling the clinical diagnosis of at least “probable bvFTD” were included [8] and all the PPA (svPPA and nfvPPA) patients had imaging-supported diagnosis [7]. FTLD-MND patients were diagnosed with probable or definite bvFTD or PPA with concomitant at least clinically possible ALS according to the revised El Escorial criteria [10]. Patients showing signs for other progressive neurodegenerative disease than FTLD or obvious damage to brain (such as stroke) were excluded from this study. The PPD group comprised patients with a severe late onset psychiatric disorder, including psychotic and mood disorders. These patients did not eventually meet at least the probable criteria (11/34 met possible bvFTD criteria but showed no progression and the behavioral disturbance was better accounted for by a psychiatric diagnosis) for any of the FTLD clinical subtypes (at the initial phase or during follow-up). The mean follow-up time for the PPD group was 16 months (median 15 months). Based on these criteria, 125 patients were eventually included in this study. At least the probable criteria for bvFTD were fulfilled in 66 patients, 16 for nfvPPA, four for svPPA, and five for FTLD-MND and 34 patients were diagnosed as having a PPD. In the FTLD group, 26 patients had a definite diagnosis due to the *C9orf72* repeat expansion (Online Resource Supplementary File 1). Out of the 34 PPD patients, psychotic disorder was present in 18/34 (53%), mood disorder in 26/34 (76%) and both mood and psychotic disorder in 10/34 (29%) of the cases. Specific diagnoses in the psychotic disorder group were: late onset schizophrenia *N* = 4, schizoaffective disorder *N* = 1, severe depression with psychotic symptoms *N* = 6, bipolar disorder with psychotic depression *N* = 1, persistent delusional disorder *N* = 4, unspecified non-organic psychosis *N* = 2, and in the mood disorder group: severe depression with psychotic symptoms *N* = 6, severe depression without psychotic symptoms *N* = 9, moderate depression without psychotic symptoms *N* = 4, and bipolar disorder *N* = 7.

For cognitive and functional evaluation, Mini-Mental State Examination (MMSE) (available from 84 participants of which 55/84 had FTLD) and Alzheimer`s disease co-operative study: activities of daily living inventory (ADCS-ADL) (available from 46 participants, of which 34/46 had FTLD) scores at baseline were tested. Follow-up scores of MMSE were available from 40 patients (30/40 had FTLD) and of ADCS-ADL from 26 patients (20/26 had FTLD), and a decline rate indicating either cognitive (MMSE) or functional (ADCS-ADL) decline was calculated for these patients (decline in points per months, higher score indicating more rapid decline). The follow-up time between the tests varied between 6 and 33 months (mean 14.5 months) in ADCS-ADL scores and between 3 and 128 months (mean 25.6 months) in MMSE scores.

### SNfL analyses (single molecule array)

SNfL was quantified according to the manufacturer’s instructions using the NfL advantage kit for the Quanterix single molecule array (Simoa, Lexington, MA, USA) [11] (Online Resource Supplementary File 1).

### Statistical analyses

Statistical analyses were performed with IBM SPSS Statistic 25. The Shapiro–Wilk test and visual inspection were used to evaluate the distribution of the data. Due to non-normal distribution of the sNfL levels, natural logarithm transformation was used to create normally distributed data (ln-NfL). General linear model, with age as a covariate, was used to compare groups as for the ln-NfL levels. Receiver operating characteristics (ROC) curve analysis was used to calculate area under curve (AUC) with 95% confidence interval and sNfL cutoff value for optimal sensitivity and specificity. Correlation of ln-NfL levels to other variables was analyzed either with Pearson’s correlation test (if the other variable was normally distributed) or Spearman’s rank correlation test (for non-normally distributed variables). *p *value ≤ 0.05 was considered as statistically significant.

## Results

The study population characteristics with mean sNfL levels in each group are described in Table 1. Within the entire cohort (FTLD and PPD combined), sNfL levels showed a positive correlation with age (*r* = 0.512, *p* < 0.0001) and did not differ between genders (*p* = 0.817). Higher sNfL levels correlated with lower MMSE scores at baseline (*r*_*s*_ = − 0.331, *p* = 0.002) and also with higher MMSE decline rate (*r*_*s*_ = 0.521, *p* = 0.001). There was no correlation between sNfL levels and baseline ADCS-ADL score (*r*_*s*_ = − 0.044, *p* = 0.773), but higher sNfl levels correlated with higher ADCS-ADL decline rate (*r*_*s*_ = 0.509, *p* = 0.009). Among the FTLD patients, the only significant correlations were the positive correlation of sNfL to age (*r* = 0.327, *p* = 0.002) and the negative correlation of sNfL to baseline MMSE score (*r*_*s*_ = − 0.268, *p* = 0.050). In the PPD group, there was a significant positive correlation with sNfL levels and age (*r* = 0.554, *p* = 0.001), and higher sNfL levels correlated with lower ADCS-ADL score at baseline (*r*_*s*_ = − 0.627, *p* = 0.029).

**Table 1:** Study population characteristics and sNfL levels in each group

Group	*N*	Age, (years), mean (SD)	Gender, %F	MMSE, mean (SD)	sNfL (pg/mL), mean (SD)
FTLD total	91	65.0 (8.7)	51.6	23.6 (4.5)	43.7 (36.3)
BvFTD	66	64.4 (9.4)	48.5	24.3 (3.6)	37.4 (32.8)
PPA	20	67.8 (6.4)	60.0	21.0 (7.1)	54.0 (36.4)
FTLD-MND	5	62.8 (4.7)	60.0	21.3 (3.8)	84.5 (49.2)
PPD total	34	55.7 (9.4)	58.8	25.4 (4.0)	15.5 (9.5)
PPD psychotic	18	57.5 (10.7)	72.2	24.4 (4.2)	18.2 (10.8)
PPD mood	26	54.0 (7.7)	50.0	25.9 (3.9)	13.2 (6.3)

**Fig. 1:**
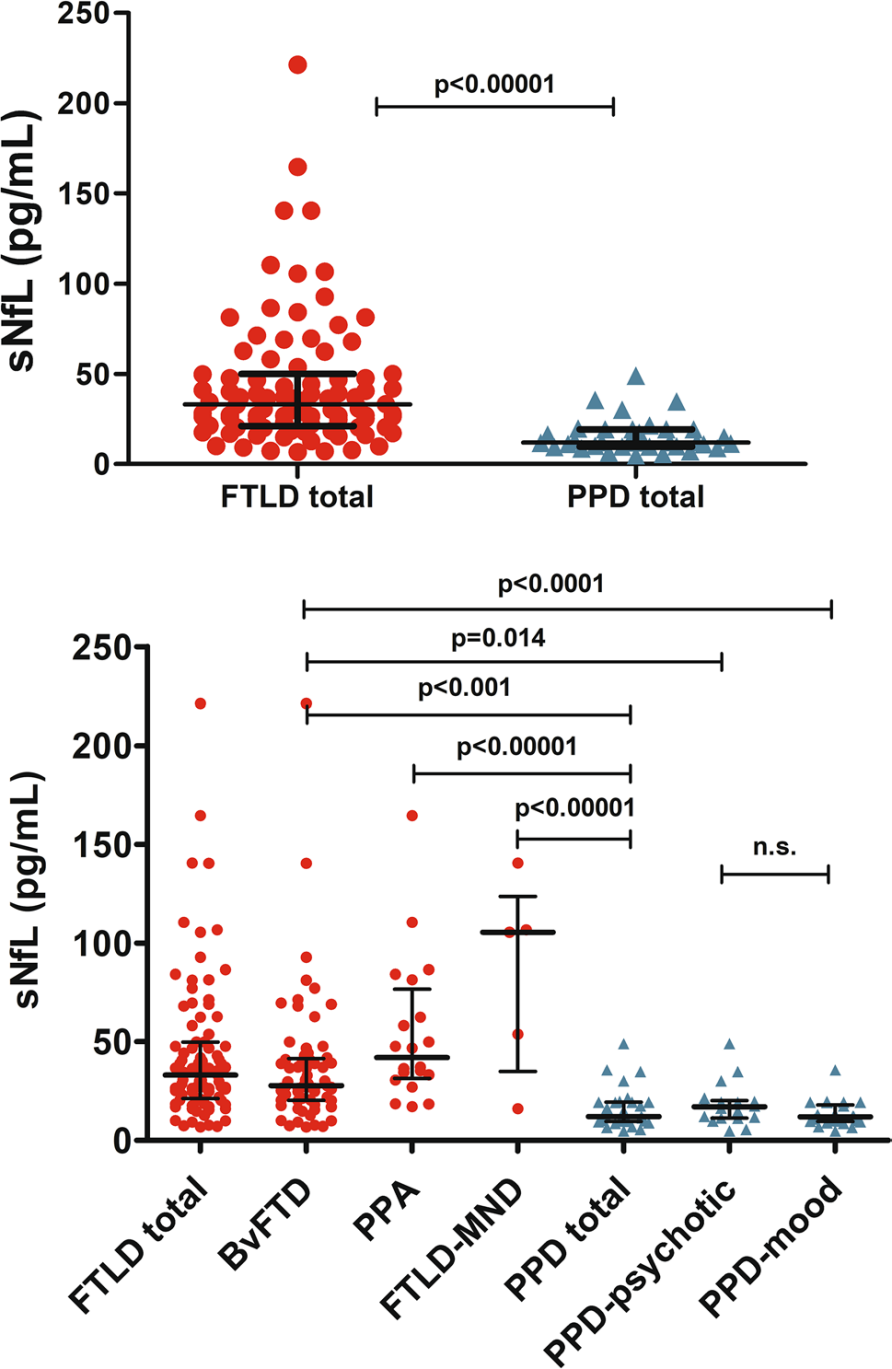
SNfL concentrations representing each case in each group with group medians and interquartile ranges (IQR). Statistical comparison was performed with general linear model (each of the comparisons made separately) with age as a covariate. *sNfL *serum neurofilament light, *FTLD *frontotemporal lobar degeneration, *bvFTD *behavioral variant frontotemporal dementia, *PPA *primary progressive aphasia, *FTLD-MND* frontotemporal lobar degeneration with motor neuron disease, *PPD *primary psychiatric disorders

Age is calculated from the date of the blood sample. In the PPD group, ten patients had both PPD psychotic and PPD mood disorder. sNfL was higher in FTLD total compared to PPD total (*p* < 0.001, adjusted for age). See Fig. 1 for subgroup comparisons

*MMSE* Mini-Mental State Examination, *sNfL* serum neurofilament light, *FTLD* frontotemporal lobar degeneration, *bvFTD* behavioral variant frontotemporal dementia, *PPA* primary progressive aphasia, *FTLD-MND* frontotemporal lobar degeneration with motor neuron disease, *PPD* primary psychiatric disorders

After adjusting for age, the levels of sNfL were higher in the total FTLD group compared to the total PPD group (β = 0.656, *p* < 0.001). Additionally, when compared individually, each of the clinical subtypes of FTLD had higher sNfL levels compared to the PPD group: bvFTD vs. PPD (β = 0.538, *p* < 0.001), PPA (svPPA and nfvPPA combined) vs. PPD (β = 0.856, *p* < 0.001) and FTLD-MND vs. PPD (β = 1.389, *p* = 0.001). There was no statistical significance in sNfL levels within the PPD group, when comparing patients based on the clinical profile (psychotic vs. mood disorder). The bvFTD group was further compared separately also to PPD subgroups (PPD psychotic and PPD mood), and sNfL levels were significantly higher in bvFTD compared to both of the PPD subgroups: bvFTD vs. PPD psychotic (β = 0.425, *p* = 0.014) and bvFTD vs. PPD mood (β = 0.632, *p* < 0.001) (Fig. 1). In the ROC analysis between the total FTLD and total PPD groups, the AUC was 0.850 (CI 95% 0.776–0.923, *p* < 0.001), and a 19.9 pg/mL sNfL cutoff level yielded discriminative sensitivity of 80% and specificity of 85%. When including only bvFTD patients and PPD patients, the values from the ROC analysis were: AUC = 0.820 (CI 95% 0.732–0.908, *p* < 0.001) and 19.9 pg/mL sNfL cutoff level resulted in 79% sensitivity and 85% specificity.

Notably, one FTLD patient with bvFTD had a substantially higher sNfL level (604 pg/mL) compared to the rest of the FTLD group (mean 43.7 pg/mL), and this outlier case was excluded from the statistical comparisons. The patient was a carrier of the *C9orf72* repeat expansion, showed wide-ranging behavioral and cognitive dysfunction, and also had psychotic symptoms and a history of prior treatment at a psychiatric ward. The patient did not recall any prior head trauma. Neurological examination showed extrapyramidal symptoms, positive snout reflex, but no signs of motor neuron disease. Also, electroencephalography (EEG) and CSF markers for Alzheimer`s disease or encephalitis were all normal. The patient died within three years after the bvFTD diagnosis. At autopsy, TDP-43 neuropathology without signs of tau or β-amyloid pathology was confirmed.

## Discussion

To date, this is the largest study reporting sNfL levels in FTLD patients compared to patients with psychiatric disorders. Although recent studies have increasingly reported elevated NfL levels in several neurodegenerative conditions, such as FTLD, AD and PD, the role of NfL in primary psychiatric disorders has remained unclear [4]. The fact that the phenotypes under the FTLD spectrum show a significant clinical overlap with psychiatric disorders [1–3], but substantially differ in the underlying pathophysiology, highlights the importance of minimally invasive pathophysiological biomarkers differentiating these two etiologies. In this present study, we have shown that sNfL levels are higher in FTLD spectrum disorder patients compared to those with PPD, which is in line with a previous report on CSF samples [12] and a recent study with serum samples [6]. As previous studies have already shown a strong correlation between serum and CSF NfL levels in various neurodegenerative disorders [13–15], our study emphasizes the utility of sNfL levels as a potential, minimally invasive differential diagnostic marker between FTLD and PPD.

Our present study revealed that a cutoff level of approximately 20 pg/mL of sNfL results in a rather high specificity (85%) in discriminating FTLD and PPD. Almost precisely similar cutoff concentration was suggested in a previous study comparing bvFTD patients and healthy controls [16]. Thus, these findings suggest that PPD may not affect the sNfL cutoff value compared to healthy controls and that the 20 pg/mL cutoff for sNfL may be used in clinical diagnostics between FTLD and non-neurodegenerative conditions (including PPD). The fact that a few patients in our PPD group showed slightly elevated concentrations (over 20 pg/mL) could indicate potential axonal damage in these patients, which might result from the underlying psychiatric disorder, or some other reason, such as minor head trauma. Interestingly, one previous study has shown slightly elevated CSF NfL levels in a subset of patients with bipolar disorder [17], but another study showed that CSF NfL levels in these patients were not associated with clinical outcomes or disease progression [18]. Although the relevance of sNfL in PPD requires further studies, it appears that the possible axonal injury for example in bipolar disorder or psychotic disorders is relatively minor and hence does not significantly affect the potential of using sNfL levels to differentiate between FTLD and PPD. Notably, the positive correlation between NfL levels and age has been observed in healthy controls as well as in patients with a neurodegenerative disease [19–21], including our present study and, therefore, there may be a need for setting age-dependent cutoff values.

Previous studies in FTLD patients have shown that NfL levels also positively correlate with disease severity and progression [22, 23]. In the present study, we found that sNfL levels correlated with the decline rates of MMSE and ADCS-ADL. However, these correlations were observed only in the entire cohort including both FTLD and PPD patients. This could be due to a small sample size, as longitudinal cognitive data were available only for 30 FTLD patients and care should be taken interpreting these non-significant results as negative. Moreover, we found that higher sNfL levels were associated with lower MMSE score at baseline in FTLD patients and lower ADCS-ADL score at baseline in PPD patients, indicating that sNfL is associated with disease severity not only in FTLD but interestingly also in PPD. We also reported a case with an exceptionally high sNfL level. This patient suffered from a rapidly progressive definite bvFTD without motor neuron disease and was first misdiagnosed with PPD. Based on this example, defining the sNfL level may be useful to shorten the diagnostic delay.

The strengths of this study include our novel and clinically relevant cohort setting with a high proportion of definite FTLD cases according to the current diagnostic criteria. Additionally, the heterogeneity in both FTLD and PPD groups in the cohort reflects real-life clinical practice. Compared to a recent paper with a nearly similar study approach (bvFTD vs. PPD) [6], in the present study, we have taken advantage of using a larger bvFTD cohort to confirm the findings related to the potential of sNfL as a discriminative biomarker between bvFTD and PPD. In addition, including also other clinical phenotypes of FTLD provides additional value, as prior psychiatric misdiagnoses are common also in the PPA phenotypes of FTLD [3]. Despite the follow-up for the PPD group, we cannot exclude that some of those patients (especially those with higher sNfL values) might eventually develop FTLD or other neurodegenerative disorder. The limitation of this study is the cohort size, especially considering the limited amount of PPD patients. Additionally, our sNfL data reflect only one cross-sectional time point at the early diagnostic phase and underscore the importance for longitudinal follow-up of the sNfL levels in the future.

In conclusion, our study shows that sNfL levels with a 20 pg/mL cutoff value are a promising blood-derived discriminating biomarker between FTLD and PPD and that the especially high levels of sNfL in FTLD are associated with a more severe disease. These findings highlight the potential of sNfL as a diagnostic and disease-monitoring tool in FTLD and, most importantly, emphasize the utility of a less invasive serum sample over lumbar puncture for biomarker analyses. Further studies are needed to confirm our findings, especially regarding the relatively low sNfL levels in PPD patients, and to determine the optimal cutoff levels for sNfL as a clinical tool for the discrimination of FTLD from other etiologies.

## Electronic supplementary material

Below is the link to the electronic supplementary material.

Supplementary file1 (PDF 150 kb)
