# Supplementary material for: Serum neurofilament light chain is a discriminative biomarker between frontotemporal lobar degeneration and primary psychiatric disorders
Source: J Neurol. 2019 Oct 8;267(1):162–7. doi: 10.1007/s00415-019-09567-8 (PMC6954884; doi:10.1007/s00415-019-09567-8)
Supplement: Supplementary file 1 — Supplementary file1 (PDF 150 kb) [file 415_2019_9567_MOESM1_ESM.pdf]

# Serum neurofilament light chain is a discriminative biomarker between frontotemporal lobar degeneration and primary psychiatric disorders

Kasper Katisko, Antti Cajanus, Olli Jääskeläinen, Aleksi Kontkanen, Päivi Hartikainen, Ville E. Korhonen, Seppo Helisalmi, Annakaisa Haapasalo, Heli Koivumaa-Honkanen, Sanna-Kaisa Herukka, Anne M. Remes, Eino Solje

**Corresponding author:**

Eino Solje, MD, PhD

University of Eastern Finland – Institute of clinical medicine, Neurology

P.O. Box 1627 (Yliopistonranta 1C) FI-70211 Kuopio, Finland

+358408425553

eino.solje@uef.fi

ORCID: 0000-0001-9940-9524

**Supplementary methods:**

## Genetic testing

The *C9orf72* repeat expansion was analyzed via the repeat-primed polymerase chain reaction assay (RP-PCR) [1], with Amplicon length analysis confirmation [2]. In total, genetic testing for the *C9orf72* repeat expansion was performed for 82% (n=103) out of the study participants. Patients without the *C9orf72* repeat expansion had less than ten repeats (N=74, including 25 PPD patients and 49 FTLD patients). Patients with the *C9orf72* repeat expansion had more than 40 repeats (N=26). None of the patients in the PPD group had the *C9orf72* repeat expansion. We did not systematically analyze other common FTLD associated genes (*GRN, MAPT*), as our previous studies partly comprising the same patients have shown that mutations in these two genes are very rare in Finnish patients [3–5].

## SNfL analyzes (Single molecule array)

All samples were collected at Kuopio University Hospital, aliquoted, and immediately frozen and stored at -80°C until analyses. Frozen serum samples were thawed in room temperature, mixed, centrifuged (10,000 x g, 5 min, RT) and transferred to a 96-well-plate. SNfL was quantified according to manufacturer's instructions using the NfL Advantage kit (REF#: 102258) for the Quanterix single molecule array (Simoa, Lexington, MA, USA) [6]. All samples were analyzed as duplicates, and mean value from the duplicates was used to determine the sNfL level for each patient.

## Supplementary references

1. Renton AE, Majounie E, Waite A, et al (2011) A hexanucleotide repeat expansion in C9ORF72 is the cause of chromosome 9p21-linked ALS-FTD. Neuron 72:257–268. https://doi.org/10.1016/j.neuron.2011.09.010

2. van der Zee J, Gijselinck I, Dillen L, et al (2013) A Pan-European Study of the C9orf72 Repeat Associated with FTLD: Geographic Prevalence, Genomic Instability, and Intermediate Repeats. Hum Mutat 34:363–373. https://doi.org/10.1002/humu.22244

3. Krüger J, Kaivorinne AL, Udd B, et al (2009) Low prevalence of progranulin mutations in Finnish patients with frontotemporal lobar degeneration. Eur J Neurol 16:27–30. https://doi.org/10.1111/j.1468-1331.2008.02272.x

4. Kaivorinne AL, Krüger J, Udd B, et al (2010) Mutations in CHMP2B are not a cause of frontotemporal lobar degeneration in Finnish patients. Eur J Neurol 17:1393–1395. https://doi.org/10.1111/j.1468-1331.2010.03028.x

5. Kaivorinne A-L, Krüger J, Kuivaniemi K, et al (2008) Role of MAPT mutations and haplotype in frontotemporal lobar degeneration in Northern Finland. BMC Neurol 8:48. https://doi.org/10.1186/1471-2377-8-48

6. Rissin DM, Kan CW, Campbell TG, et al (2010) Single-molecule enzyme-linked immunosorbent assay detects serum proteins at subfemtomolar concentrations. Nat Biotechnol 28:595–599. https://doi.org/10.1038/nbt.1641
